# Presynaptic Activity of an Isolated Fraction from Rhinella schneideri Poison

**DOI:** 10.15171/apb.2018.060

**Published:** 2018-08-29

**Authors:** Sandro Rostelato-Ferreira, Cháriston André Dal Belo, Pedro Ismael da Silva Junior, Stephen Hyslop, Léa Rodrigues-Simioni, Thomaz Augusto Alves Rocha-e-Silva

**Affiliations:** ^1^Departamento de Farmacologia, Faculdade de Ciências Médicas, Universidade Estadual de Campinas (UNICAMP), Zip Code 13083-970, Campinas, SP, Brazil.; ^2^Instituto de Ciências da Saúde, Universidade Paulista (UNIP), Zip Code 18087-101, Sorocaba, SP, Brazil.; ^3^Centro de Ciências Rurais de São Gabriel, Universidade Federal do Pampa (UNIPAMPA), São Gabriel, RS, Brazil.; ^4^Laboratório Especial de Toxinologia Aplicada, Instituto Butantan, São Paulo, SP, Brazil.; ^5^School of Medicine Faculdade Israelita de Ciências da Saúde Albert Einstein, São Paulo, SP, Brazil.

**Keywords:** Neurotransmitter release, Presynaptic toxin, Neuromuscular junction, Isolated fraction

## Abstract

***Purpose:*** Rhinella schneideri is a toad found in many regions of the South America. The poison of the glands has cardiotoxic effect in animals and neuromuscular effects in mice and avian preparation. The purpose of this work was to identify the toxin responsible for the neuromuscular effect in avian and mice neuromuscular preparation.

***Methods:*** The methanolic extract from R. schneideri poison was fractioned by reversed phase HPLC. The purity and molecular mass were determined by LC/MS mass spectrometry. Chick biventer cervicis and mouse phrenic-nerve diaphragm were used as neuromuscular preparations to identify the toxin.

***Results:*** The purification resulted in 32 fractions, which 4 of them were active in neuromuscular preparation. The toxin of fraction 20 were chosen for better reproducibility of the whole extract activity and its molecular mass was 730.6 Da. The toxin produced facilitation of the muscle contraction followed by a complete neuromuscular blockade in chick biventer cervicis preparation in 90 min without interfering with the exogenous response to ACh and KCl. The quantal content was increased from 128 ± 13 (control) to 216 ± 44 (after 5 min and sustained until 60 min) in the presence of the toxin.

***Conclusion:*** In conclusion, our results demonstrated that the neuromuscular action of the poison of Rhinella schneideri is a multitoxin effect. More, the present work first isolated a 730.6 Da toxin that better represent the whole poison neuromuscular effect, to which is attributed a presynaptic action in avian and mouse neuromuscular preparation.

## Introduction

*Rhinella schneideri* is a commonly found toad in South American countries. This amphibian poison is produced by a large post-orbital parotid glands and secretions help to protect against predators. This defense mode is considered as a passive mechanism of such animals since they lack spines, nails, or sharp teeth.^1^

The poison is composed by biogenic amines, steroids, alkaloids, peptides and proteins.^2^ The steroids are responsible for accelerating the heart rate of affected animals, but also induces apoptosis and hallucinogenic effects, while peptides and proteins are believed to improve toad defense against microorganisms.^3^

The methanolic extract of *R. schneideri* poison has shown to act presynaptically in neuromuscular preparations, albeit with discrepancies. In chick biventer cervicis *in vitro* nerve-muscle preparation, *the* poison leads to neuromuscular blockade by inhibiting acetylcholine release, without interfering with muscle integrity.^4^ On the other hand, it increases the release of the neurotransmitter facilitating the neurotransmission in mice hemi-diaphragm preparation.^5^

Isolated compounds from *R. schneideri* poison have been studied in haematological models and showed ability of reducing the complement hemolytic activity of the classical/lectin pathways after preincubation with normal human serum.^6^ The acetylated bufadienolides, major toxins from toads, showed lesser peripheral inhibitory activity of blood lymphocytes than their precursors, suggesting that chemical modifications on such compounds can play an important role on the modulation of their cytotoxic profile.^7^ Despite those results, there is no study on neuromuscular junction of any toad poison isolated compound.

Among these previous results on neuromuscular junction, we considered the importance to purify the substance(s) capable to produce the neuromuscular effects. In this work, the aim was to identify the toxin(s) responsible by affecting the neurotransmission in avian and mice neuromuscular preparation.

## Materials and Methods

### Material

The poison was collected by manual compression of the large post-orbital parotid toad glands. The amount of 2 g was immersed in 50 mL of methanol during three days, at room temperature, lyophilized in a SpeedVac centrifuge^8^ and stored at -20°C. The lyophilized methanolic extract was dissolved in Tyrode solution prior to use.

### Animals

Male Swiss mice (25-30g) were obtained from the Multidisciplinary Center for Biological Investigation (CEMIB/UNICAMP), and male HY-LINE W-36 chicks (4-8 days old) were supplied by Granja Globo Aves Agrovícola Ltda (Mogi Mirim, SP, Brazil). Animals were housed at 23 ± 3°C under a 12h light/dark cycle with free access to food and water.

### Purification of the methanolic extract

Ten milligrams of the methanolic extract of *R. schneideri* poison was dissolved in 40 µL of amonium bicarbonate completing the volume to 100 µL using trifluoracetic acid (TFA) 0.1% and centrifugated (14000 g, 3 min, 4°C) to remove the insoluble material. The supernatant was used for purification.

The methanolic extract was fractioned by HPLC on a reversed phase Phenomenex Luna PFP (250 x 4.6 mm) with TFA 0.1% + acetonitrile (ACN) 10% as mobile phase and ACN 90% + 0.1% TFA as eluent. Fractions were eluted with a linear gradient 0-65% of eluent. The outflow was monitored at 214 e 280 nm. The fractions were manually collected and then lyophilized.^9^

### MALDI-TOF mass spectrometry

The purity and molecular mass of toxin from methanolic extrat from *R. schneideri* poison were determined by mass spectrometry. The fraction samples (0.5 ml) were spotted onto the sample slide and dried on the bench and crystallized with 0.5 ml of matrix solution [5 mg/ml (w/v) CHCA (a-cyano-4-hydroxycinnamic acid), in 50% acetonitrile and 0.1% TFA] (Sigma). The sample was analyzed on an Ettan MALDI-ToF/Pro spectrometer (Amershan Biosciences) operating in reflectron mode.^9^

### Chick biventer cervices preparation

Male chicks were euthanized with isoflurane overdose. The biventer cervicis muscles^10^ were mounted under a tension of 1 g/0.5 cm in a 5 mL organ bath containing warmed (37°C), aerated (95% O_2_ + 5% CO_2_) Krebs solution of the following composition (mM): NaCl 118.7, KCl 4.7, CaCl_2_ 1.8, NaHCO_3_ 25, MgSO_4_ 1.17, KH_2_PO_4_ 1.17 and glucose 11.65, pH 7.5. A bipolar platinum ring electrode was placed around the tendon within which runs the nerve trunk supplying the muscle. Field stimulation (0.1 Hz, 0.2 ms, 4-6 V) was performed with a Grass S48 stimulator (Astro-Med Inc., W. Warwick, RI, USA). Muscle contractions and contractures were recorded isometrically via a force-displacement transducer (Load Cell BG- 10GM, Kulite Semiconductor Products Inc., NJ, USA) coupled to a Gould model RS3400 physiograph via a Gould universal amplifier (Gould Inc., Cleveland, OH, USA).

Contractures to exogenously acetylcholine (ACh) and KCl were obtained in the absence of field stimulation prior to the addition of toxin and at the end of the experiment, as a test for the presence of myotoxic and neurotoxic activities.^11^ The preparations were allowed to stabilize for at least 20 min before the addition of ACh, KCl or isolated toxin to the bath. Twitch-tension responses to the toxin were monitored for up to 90 min, depending on the neuromuscular blockade.

The concentration was chosen based in the profile of neuromuscular response, similar to obtained date in previous studies with the methanolic extract only. The concentration of the toxin (3 µg/mL) used here presented an effect equivalent to 30% of the methanolic extract of *R. schneideri* poison.^4^

### Mouse phrenic-nerve diaphragm preparation – Quantal content

For electrophysiological technique the mice were euthanized by isoflurane overdose a confirmed by exsanguination. The phrenic nerve-hemidiaphragm (PND) preparations were dissected according to Bülbring.^12^ The preparations were incubated for 60 minutes with 15 µg/mL of the isolated toxin in Tyrode solution (composition in mM: NaCl 137, KCl 2.7, CaCl_2_ 1.8, MgCl_2_ 0.49, NaH_2_PO_4_ 0.42, NaHCO_3_ 11.9 and glucose 11.1; pH 7.5 at 37 °C) and constantly aerated by carbogen (95 % O_2_ – 5 % CO_2_), while control samples were exposed to Tyrode alone (n = 4 per group). Quantal content was examined as described by Banker, Kelly and Robbins^13^ using the cut muscle technique to abolish the end-plate potential which involves the activation of muscle Na^+^ channels. The recordings were obtained 0, 15, 30 and 60 min after the addition of Tyrode solution alone (control) or isolated toxin (15 mg/mL; concentration equivalent to 30% of the effect of the methanolic extract, like cited above).

### Statistical analysis

The results were reported as the mean ± S.E.M. Student’s *t*-test and repeated-measures analysis of variance (ANOVA) were used for statistical comparison of the data, with a value of* P* <0.05 indicating significance. All data analyses were done using Microcal OriginPro 8 software.

## Results

### Purification of the toxin

**Figure 1:**
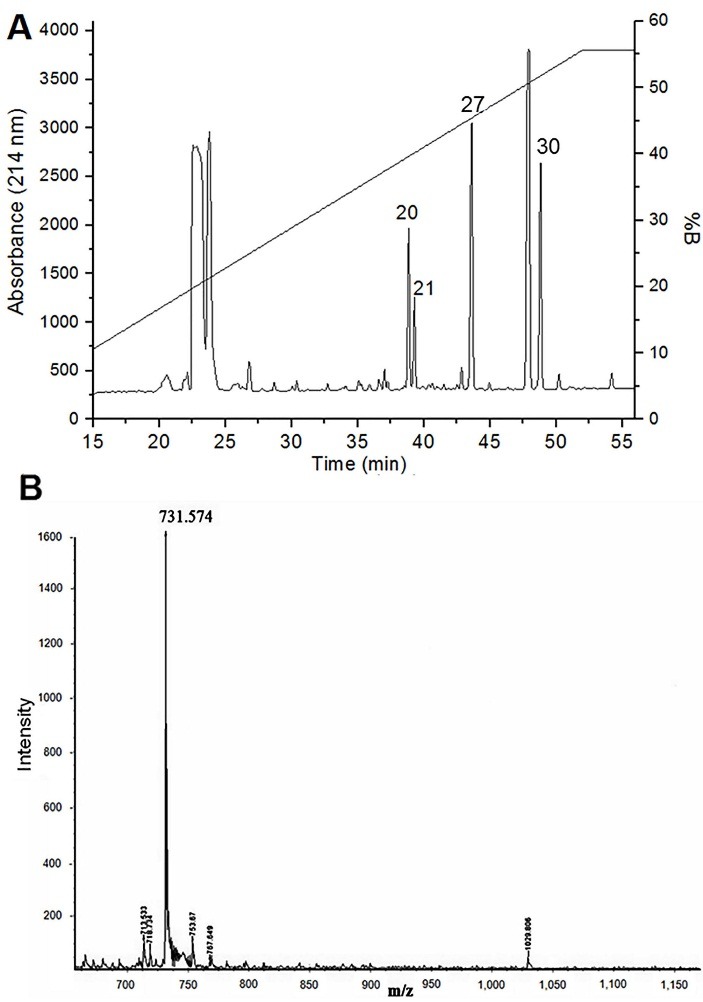
Purification of toxin from the *Rhinella schneideri* poison by reversed phase HPLC. (A) Chromatogram of RP-HPLC was performed on a reversed phase Phenomenex Luna Pentafluorophenyl (250 x 4.6 mm) with TFA 0.1% + acetonitrile (ACN) 10% as mobile phase and ACN 90% + 0.1% TFA as eluent. Fractions were eluted with a linear gradient 0-65% of eluent. In (A) the elution profile was also monitored at 214 nm and showed essentially the same result as at 280 nm (data not shown). The profile matches to 1 mg of the methanolic extract, and numbered fractions presented effect on neuromuscular preparation. (B) MALDI-TOF mass spectrometry of isolated toxin showing the purity and mass (730.6 Da) of the toxin

The purification of the methanolic extract resulted in seven major and several minor peaks (Figure 1A), reaching 32 fractions total. All the major peaks were tested in neuromuscular preparation and the fractions 20, 21, 27 and 30 presented neuromuscular activity.

### Mass spectrometry

Fraction 20 was analyzed by MALDI-TOF mass spectrometry resulting in M/Z 731.574. The molecular mass of the isolated toxin was determined as 730.6 Da (Figure 1B).

### Chick biventer cervicis preparation

**Figure 2:**
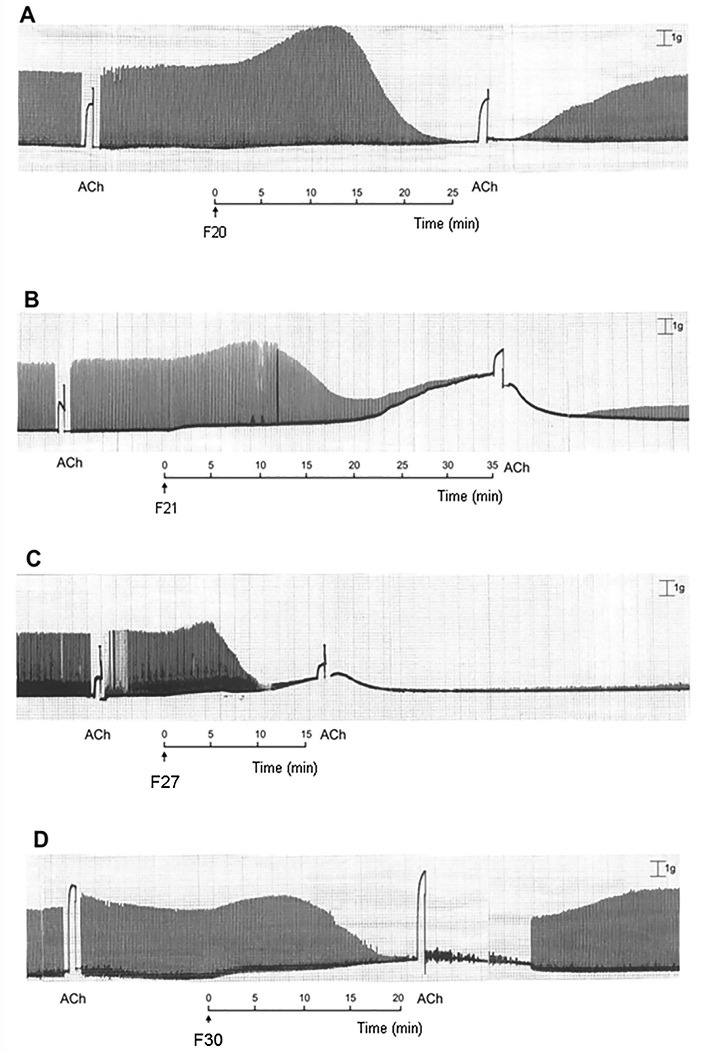
Myographic record of chick biventer cervicis preparation under indirect stimulation. The preparations were treated with the fractions 20 (A), 21 (B), 27 (C) and 30 (D). Note that all the fractions induced a facilitation effect followed by neuromuscular blockade. Tension: 1 g

These fractions were capable to produce a facilitation followed by neuromuscular blockade, without interfering with post synaptic responses (Figure 2). Fraction 20 was the toxin capable to reproduce a similar neuromuscular effect of that obtained with the methanolic extract of *R. schneideri* poison, previously observed.

**Figure 3:**
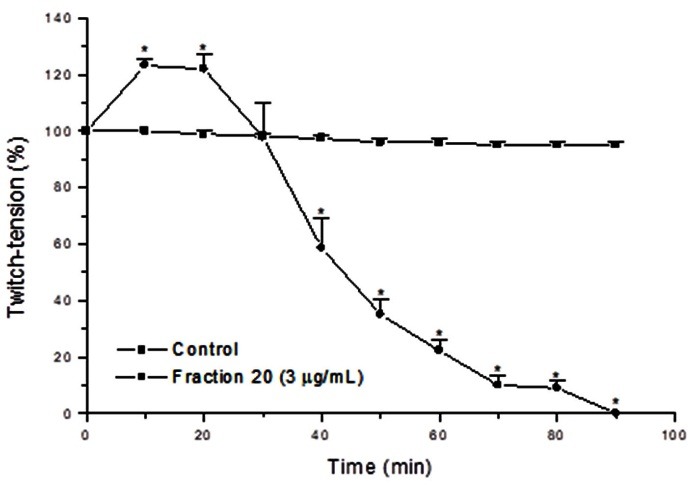
Graphic representing neuromuscular response of chick biventer cervicis under indirect stimulation. The isolated toxin (3 µg/mL) produced a facilitation effect followed by neuromuscular blockade. The points represent the mean ± SEM of 4 experiments. *p < 0.05 compared to the corresponding responses in the control group

The isolated toxin (fraction 20) at 3 µg/mL induced a complete neuromuscular blockade preceded by a facilitation of neurotransmission (Figure 3). Hence, the blockade observed was biphasic, with an increase of the twitch-tension followed by complete neuromuscular blockade. The time delay to reach neuromuscular blockade was similar to previous results of crude methanolic extract of *R. schneideri* poison.

**Figure 4:**
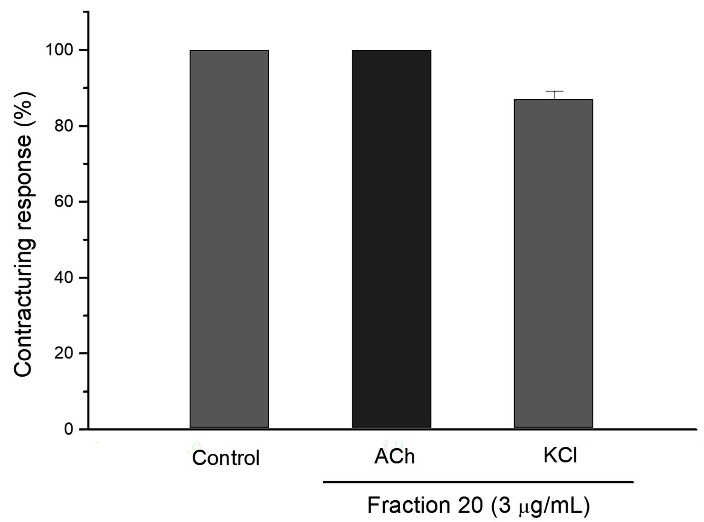
Graphic representing the contracturing response of chick biventer cervicis preparation under exogenous addition of ACh and KCl. Note that the isolated fraction did not significantly alteration after the toxin addition. The columns represent the mean ± SEM of 4 experiments

The neuromuscular blockade did not significantly affect the contractures to exogenous ACh and KCl (Figure 4), attesting the integrity of the muscle receptors and membrane.

### Quantal content - Electrophysiological technique

**Figure 5:**
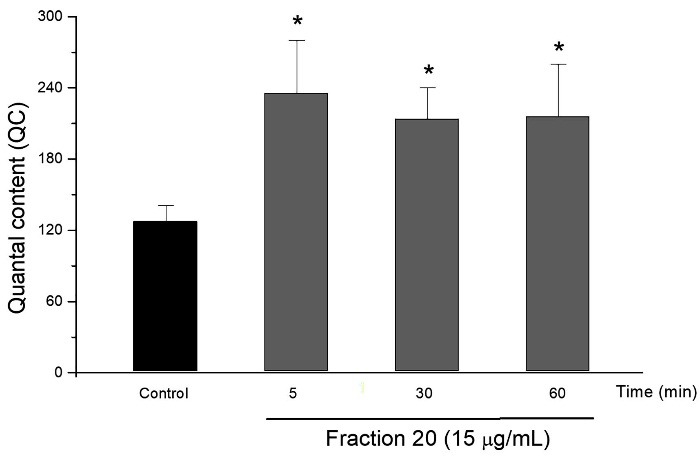
Graphic representing the alteration of quantal content value (end-plate potential) of mouse phrenic-nerve diaphragm preparation. The isolated toxin (15 µg/mL) produced a significantly increase of the quantal content value that was maintained during 60 min of observation. The columns represent the mean ± SEM of 4 experiments. *p < 0.05 compared to the corresponding responses in the control group.

The isolated toxin (15 µg/mL) significantly increased the quantal content (increase in the release of acetylcholine from the nervous terminal) within five minutes, from 128 ± 13 (control) to 234 ± 44 (5 min). The increase of the quantal content was maintained until the end of the observation (Figure 5). All the values observed are shown in the Table 1.

## Discussion

Cutaneous secretions of toad species are an important source of compounds with interest to biopharmacological properties. Previous studies showed that methanolic extract of *R. schneideri* poison contains substances capable of affecting neurotransmission in avian and mammalian preparations. The extract was able to cause neuromuscular blockade in avian preparation and sustained muscle facilitation that apparently resulted from enhanced presynaptic neurotransmitter release since electrophysiological measurements indicated an increase in MEPP frequency.^4,5^

**Table 1:** Effect of isolated toxin on the quantal content

**-**	**Quantal content**
**Control**	128±13
**5 min**	234±44*
**30 min**	214±26*
**60 min**	216±44*

*p < 0,05 comparing to control

Zelnik, Ziti and Guimarães^14^ made a chromatographic study of the bufadienolides isolated from the poison of the parotid glands of *Bufo paracnemis* (former *R. schneideri*) Lutz 1925 and identified the compounds: γ-sitosterol, argentinogenin, bufalin, bufotalinin, gamabufotalin, hellebrigenin, hellebrigenol, resibufogenin and telocinobufogenin. But few studies have described the isolation and biological effects of molecules of the *R. schneideri* poison,^6,7,15^ and there is limited information about identification of substances from toad poison, as well as, their pharmacological activities. Among them, Anjolette et al.^6^ isolated and partially characterized the function and structure of the first bufadienolide (6,000 Da) with inhibitory action over chymotrypsin, isolated from *R. schneideri* poison. Souza-Filho et al.^15^ isolated a galectin, from *R. schneideri* poison, that presented an anti-inflammatory activity and slight toxic effects in mice.

Cunha-Filho et al.^7^ isolated ten bufadienolides from *R. schneideri* poison and showed that the compounds 3beta-acetoxy-marinobufagin, 3beta-acetoxy-bufalin, 3beta-acetoxy-telocinobufagin, and 3beta-acetoxy-20S, 21R-epoxymarinobufagin (~ 400 Da) presented results suggesting that chemical modifications on such compounds can play an important role on the modulation of their cytotoxic profile.

In this work, a toxin (730.6 Da) was identified and evaluated under neuromuscular activity that showed a presynaptic effect in avian and mice neuromuscular preparation. The results showed a toxin isolated with a neuropharmacological effect under avian and mice neuromuscular preparation, and the most important, maintaining the muscle integrity. In avian preparation was observed a biphasic blockade, with an increase in twitch-tension followed by complete neuromuscular blockade. These results were similar to those obtained using the methanolic extract from *R. schneideri* toad poison.^5^ A similar biphasic response has also been observed in mammalian preparations incubated with snake venom presynaptic neurotoxins such as β-bungarotoxin, notexin^16^ and crotoxin.^17^ However, the biphasic activity seen here was not accompanied by alterations in the muscle contractures to exogenous ACh or KCl. Dal Belo *et al.*^18^ observed a similar increase in the quantal content of mouse neuromuscular preparations after ten-minute incubation with MiDCA1, a toxin isolated from coral snake (*Micrurus dumerilli carinicauda*) venom and concluded that a presynaptic action was involved.

Inhibitors of Na+/K+/ATPase pump, like digoxin and ouabain, are able to potentialize the release of the neurotransmitter and facilitate spontaneous and evoked release of Ach, thereby increasing the frequency of miniature end-plate potential and amplitude of single end-plate potentials.^19,20^ The methanolic extract from *R. schneideri* poison has shown a digoxin-like effect, and the ouabain (1 µM) is able to inhibit this effect.^5^ Herein, the isolated toxin clearly presented a digoxin-like effect, by causing a maintained increase of the quantal content,^5^and neuromuscular blockade in avian preparation,^4^without interfering with the muscle integrity.

Taken together, the results observed here indicate that the isolated toxin of the *R. schneideri* poison was able to reproduce the effects observed with the methanolic extract using avian and mouse neuromuscular preparation.

## Conclusion

In conclusion, our results demonstrated that the neuromuscular action of the poison of *Rhinella schneideri* is a multitoxin effect. More, the present work first isolated a 730.6 Da toxin that represent the whole poison neuromuscular effect, to which is attributed a presynaptic action in avian and mouse neuromuscular preparation.

## Acknowledgments

We thank Gildo B. Leite for technical assistance. This work was supported by Conselho Nacional de Desenvolvimento Científico e Tecnológico (CNPq) (201342/2010-3) and Fundação de Amparo à Pesquisa do Estado de São Paulo (Fapesp) (08/54050-0).

## Ethical Issues

The animal experiments were approved by the Institutional Committee for Ethics in Animal Use (CEUA/UNICAMP, protocol no. 1552-1) and were in accordance with the ethical guidelines established by the Brazilian Society of Laboratory Animal Science (SBCAL, formerly the Brazilian College of Animal Experimentation - COBEA).

## Conflict of Interest

The authors have no conflicts of interest.
